# Previous Exposure to Advanced Therapies in Acute Severe Ulcerative Colitis: A New Risk Factor for Colectomy?

**DOI:** 10.1093/crocol/otaf054

**Published:** 2025-09-14

**Authors:** Maria Paz Gimenez Villamil, Paulo Gustavo Kotze

**Affiliations:** Health Sciences Postgraduate Program, Pontifícia Universidade Católica do Paraná (PUCPR), Curitiba, Brazil; IBD Outpatient Clinics, Colorectal Surgery Unit, Cajuru University Hospital, Pontifícia Universidade Católica do Paraná (PUCPR), Curitiba, Brazil; Health Sciences Postgraduate Program, Pontifícia Universidade Católica do Paraná (PUCPR), Curitiba, Brazil; IBD Outpatient Clinics, Colorectal Surgery Unit, Cajuru University Hospital, Pontifícia Universidade Católica do Paraná (PUCPR), Curitiba, Brazil

The therapeutic landscape for ulcerative colitis (UC) has undergone significant changes over recent decades, particularly with the introduction of biologic agents and small molecules that have become increasingly available and are now widely used in outpatient settings.^1^ Although the broad availability of effective therapeutic options has led to a paradigm shift in the treatment outcomes and clinical management of inflammatory bowel disease (IBD), a subset of patients still either fail to respond or eventually loses responsiveness to therapy.^2^

Acute severe ulcerative colitis (ASUC) remains a common medical emergency, with up to 25% of patients experiencing at least one severe episode during their lifetime.^3^ Despite therapeutic advances, morbidity remains substantial, with an estimated mortality rate of 1% and colectomy rates reaching up to 30% during the initial hospitalization, and a cumulative rate of 40% over the disease course.^3–5^ Risk factors for colectomy include young age at diagnosis, prior hospitalizations, extensive colitis, severe endoscopic activity, hypoalbuminemia, anemia, and elevated inflammatory markers.^6^ More recently, prior exposure to anti-TNF therapy has also been identified as a potential risk factor.^7^ However, whether failure of other advanced therapies—despite differing mechanisms of action—also increases the risk of colectomy remains to be elucidated.

While novel therapies have revolutionized the management of UC, they bring forth new challenges and uncertainties, particularly in relation to disease progression and long-term outcomes.^8^^,^^9^ It is well established that prior exposure to advanced therapies is associated with a reduction in the effectiveness of subsequent treatments. The exact mechanism underlying this phenomenon—observed across different therapeutic classes—remains unclear. Potential contributing factors include prolonged disease duration, which may promote fibrosis, and the dynamic nature of inflammatory pathways, which may evolve over time.^10^

In this issue of *Crohn’s and Colitis 360*, Al-Bawardy et al.^11^ present a multicenter, retrospective study evaluating the impact of previous outpatient advanced therapy exposure (ATE) on hospitalization outcomes in 370 adult patients with ASUC. The authors report a significantly higher colectomy rate among ATE patients compared to therapy-naïve patients (25% vs 9%, *P* < 0.001). Notably, the length of hospitalization and the need for rescue medical therapy did not differ significantly between the two groups. These findings suggest that prior exposure to advanced therapies may be a marker of more refractory or severe disease, thereby contributing to higher colectomy rates.

ASUC necessitates immediate hospitalization, with intravenous corticosteroids as the cornerstone of initial treatment. Nevertheless, approximately 30% of patients fail to respond to standard therapy.^12^^,^^13^ While the mechanisms underlying reduced treatment efficacy in IBD remain poorly understood, early identification of patients unlikely to respond could facilitate more personalized treatment approaches, especially in refractory cases.^2^

The study by Al-Bawardy et al. raises the possibility that an early need for advanced therapies may reflect a more aggressive disease phenotype, thereby predisposing patients to surgical intervention. This is supported by their multivariable analysis, which identified low serum albumin as the only independent predictor of colectomy, further emphasizing the severity of illness in this cohort.

Although prior research has demonstrated that advanced therapies reduce UC flare-ups and hospitalizations, this study reveals a more nuanced reality. Its findings could influence clinical decision-making in both outpatient and inpatient settings, and suggest the need to refine monitoring and treatment strategies for patients who have failed multiple therapies. Emphasizing the use of biomarkers to predict treatment response may prove especially valuable. At the very least, heightened vigilance is warranted for patients with prior therapeutic failures. Early involvement of surgical teams during hospital admissions may help better define the optimal timing for colectomy, which remains a key component of multidisciplinary ASUC management.^14^

Despite its strengths, including a large sample size, the study’s retrospective nature and its conduct across only two centers introduce potential biases. Furthermore, the specific medications to which patients in the ATE group were previously exposed were not clearly detailed. Future prospective studies are needed to validate whether prior exposure to advanced therapies constitutes an independent risk factor for colectomy and to explore the underlying biological mechanisms. Biomarkers such as C-reactive protein and fecal calprotectin could be instrumental in guiding rescue therapy, particularly in patients with multiple prior treatment failures.^2^^,^^15^

In summary, as the therapeutic arsenal for UC continues to expand, an increasing proportion of ASUC patients will likely present with histories of multiple therapy exposures and failures. Defining the role of rescue treatments—including cyclosporine, infliximab, and JAK inhibitors—in this emerging patient subset is a critical research priority. Focused attention on this population, alongside efforts to identify predictive biomarkers and integrate novel therapeutic strategies, may ultimately enhance both short- and long-term outcomes for patients with UC.

## Conflicts of Interest

M.P.G.V. has no conflicts of interest. P.G.K. is speaker and consultant for AbbVie, Johnson & Johnson, Pfizer, and Takeda. He had received scientific grants from Pfizer and Takeda. P.P.G.K. holds the position of Associate Editor for *Crohn’s & Colitis 360* and has been recused from reviewing or making decisions for the article.
